# Complete Genome Sequence of *Flavobacteriales* Bacterium Strain UJ101 Isolated from a Xanthid Crab

**DOI:** 10.1128/genomeA.01551-16

**Published:** 2017-02-02

**Authors:** Jhung-Ahn Yang, Kae Kyoung Kwon, Hyun-Myung Oh

**Affiliations:** a Department of Marine-Bio Convergence Science, Specialized Graduate School Science and Technology Convergence, Pukyong National University, Busan, Republic of Korea; b Marine Biotechnology Research Division, Korea Institute of Ocean Science and Technology, Ansan, Republic of Korea

## Abstract

*Flavobacteriales* bacterium strain UJ101 was isolated from a xanthid crab species collected from the East Sea of Korea. Here, we report the complete genome sequence of strain UJ101 for the study of major metabolic pathways related to microbial species from marine invertebrate species.

## GENOME ANNOUNCEMENT

Flavobacteria are composed of a large variety of bacterial groups and have been isolated in many environments, including soil, marine water, plants, and animal gut. They play an important role in bacterial populations in aquatic and terrestrial environments, accounting for more than 20% of microbial communities (1). Some flavobacterial species are also responsible for severe fish disease (2), promoting the protection and growth of plants and animals (3), and the biological purification of soil or marine sediments (4, 5). Here, we report the complete genome sequence of *Flavobacteriales* bacterium strain UJ101, whose proper taxonomic validation is under way. A xanthid crab species, *Atergatis reticulatus* (family* Xanthidae*), was collected around the Uljin Nuclear Plant on the east coast of the Republic of Korea. The crab sample was processed by previous methods (6), which resulted in the isolation of strain UJ101.

This whole-genome sequence was determined using the PacBio RSII long-read sequencing method and *de novo* assembly according to previous methods (7). The sequencing information was assembled with HGAP, Celera Assembler, and Quiver, with a genome coverage of 1,063×. Open reading frames were identified with Glimmer version 3 (8), and each sequence was annotated with Blast2Go. All protein sequences were identified against the NCBI database and analyzed using the RAST server (9) and the CLGenomics program (http://www.chunlab.com), as done in a previous study (7).

The genome of strain UJ101 consists of a circular chromosome of 3,074,209 bp with a 30.74% G+C content. This genome contains 2,740 total genes, among which are 2,684 protein-coding regions and 197 protein-encoding genes with predicted function only. This strain contains 46 sets of tRNA genes and nine rRNA genes (i.e., 16S-23S-5S). There was no existing clustered regularly interspaced short palindromic repeat (CRISPR) sequence in the chromosomal analysis of strain UJ101.

Metabolic analysis was impaired in the Entner-Doudoroff pathway. Tricarboxylic acid cycle and glyoxylate cycle were conserved, while carbon fixation and metabolism were mostly lacking, except for formaldehyde dehydrogenase. Degradation enzymes present included eight glycosyl transferases, three amylases, and eight peptidases. Biosynthetic enzymes for lysine, tryptophan, phenylalanine, and tyrosine were also impaired. Alcohol and/or organic acid fermentation could not be expected.

The remarkable metabolic pathway was nitrogen, starch degradation, and folate metabolism. This strain was expected to use nitroalkane for the nitrogen cycle. Strain UJ101 was rich in amylase genes and related sequences to make use of trehalose and starch decompositions. Folate biosynthesis-related genes were represented, except for the enzyme producing dihydroneopterin (2-amino-4-hydroxy-6-hydroxymethyldihydropteridine diphosphokinase [EC:2.7.6.3]).

### Accession number(s).

The complete genome sequence of UJ101 has been deposited in GenBank under the accession number CP016269.
